# Supplementary material for: Comparative proteomics reveals that fatty acid metabolism is involved in myocardial adaptation to chronic hypoxic injury
Source: PLoS One. 2024 Jun 17;19(6):e0305571. doi: 10.1371/journal.pone.0305571 (PMC11182518; doi:10.1371/journal.pone.0305571)
Supplement: S1 File — (PDF) [file pone.0305571.s001.pdf]

AZENTA
LIFE SCIENCES

Cell Line Authentication Report

GENEWIZ, Inc.

C3 Building, 218 Xinghu Road

Suzhou Industrial Park, 215123

Suzhou, China

Tel: 400-8100-669

www.genewiz.com

AZENTA
LIFE SCIENCES

Cell Line Authentication Report

Customer: Hu Chen

Quotation Number: 80-654000427

Completion Date: 31/1/2024

1. **Sample ID: MCM**
2. **Original Material: DNA**
3. **Methods:**

1).Genomic DNA was extracted from the cell pellets provided by the customer.

2).Samples, together with positive and negative control were amplified using GenePrint 10 System (Promega).

3). Amplified products were processed using the ABI3730xl Genetic Analyzer.

4). Data were analyzed using GeneMapper4.0 software and then compared with the ATCC, DSMZ, JCRB and RIKEN databases for reference matching.

4. **Results:**

1) 10 Loci STR Profile：

Genetic Site	Cell Bank information		Customer sample	
(Locus)	HL1		MCM	
4-2	17	17	17	17
5-5	15	15	15	15
6-4	18	18	18	18
6-7	12	12	12	12
9-2	15	15	15	15
12-1	16	16	16	16
15-3	25	25	25	25
18-3	16	16	16	16
X-1	25	26	25	26
D4S2408				
Percent match between the sample and the database profile： 100%				

Summary:

Your cell line is considered to be “identical” to the reference cell line in the Cell Bank STR database, as the STR profile yields a 100% match.

Notes：

1. P=100% x (2xM)/N; M=18, N=36 P=100% x (2x18)/36=100%

M: number of the matching peaks; N: number of all peaks

2. Based on the ANSI Standard, cell lines with ≥80% match are considered to be related; i.e., derived from a common ancestry. Cell lines with between a 55% to 80% match require further profiling for authentication of relatedness.

3. The short tandem repeat (STR) profile generated by GENEWIZ Inc. is indicative only of the sample sent to GENEWIZ Inc. at the time it was sent. This data and analysis are for research use only.

2) Electrophoretogram

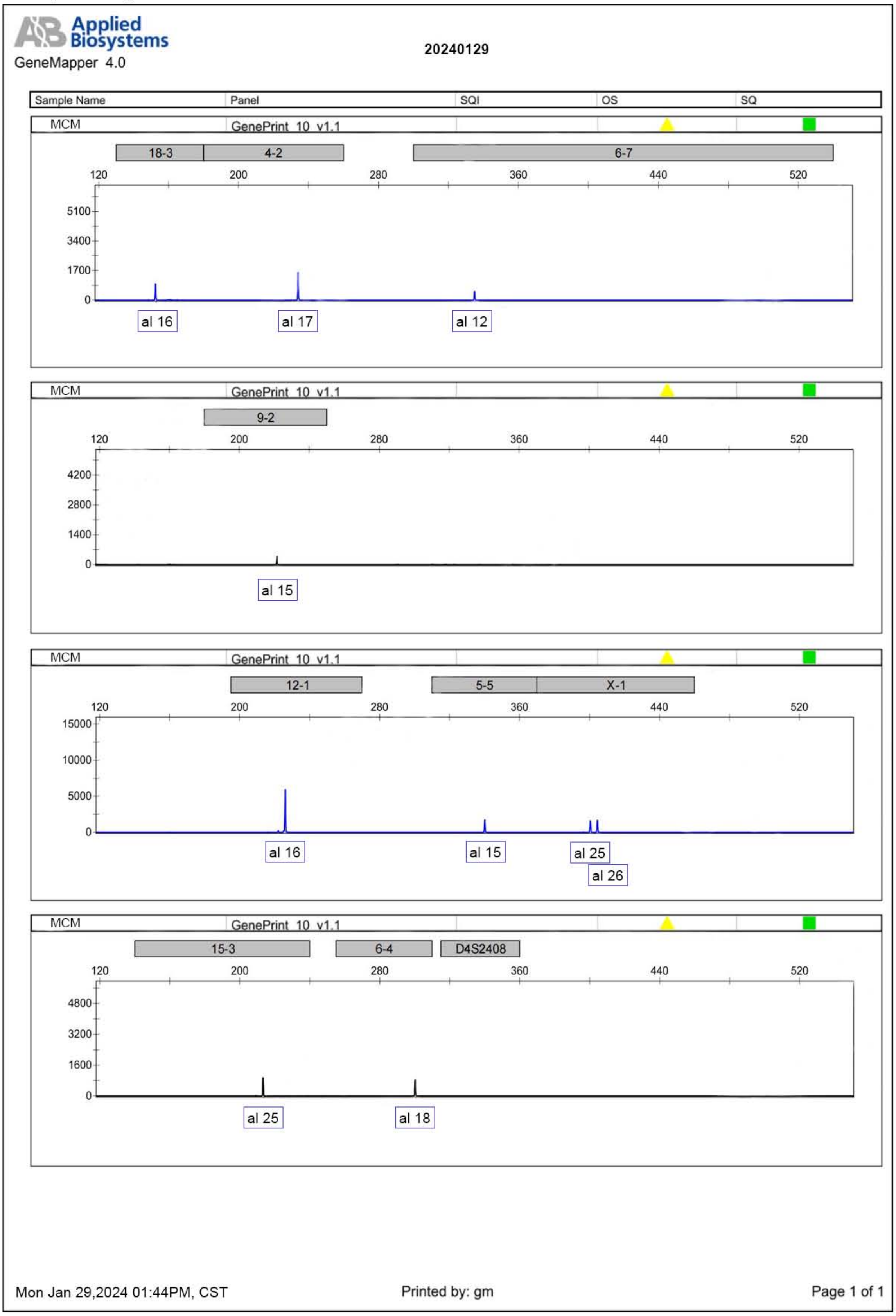

Note：Raw data in appendix
