# Supplementary material for: Comparative proteomics reveals that fatty acid metabolism is involved in myocardial adaptation to chronic hypoxic injury
Source: PLoS One. 2024 Jun 17;19(6):e0305571. doi: 10.1371/journal.pone.0305571 (PMC11182518; doi:10.1371/journal.pone.0305571)
Supplement: S1 Fig — (DOCX) [file pone.0305571.s002.docx]

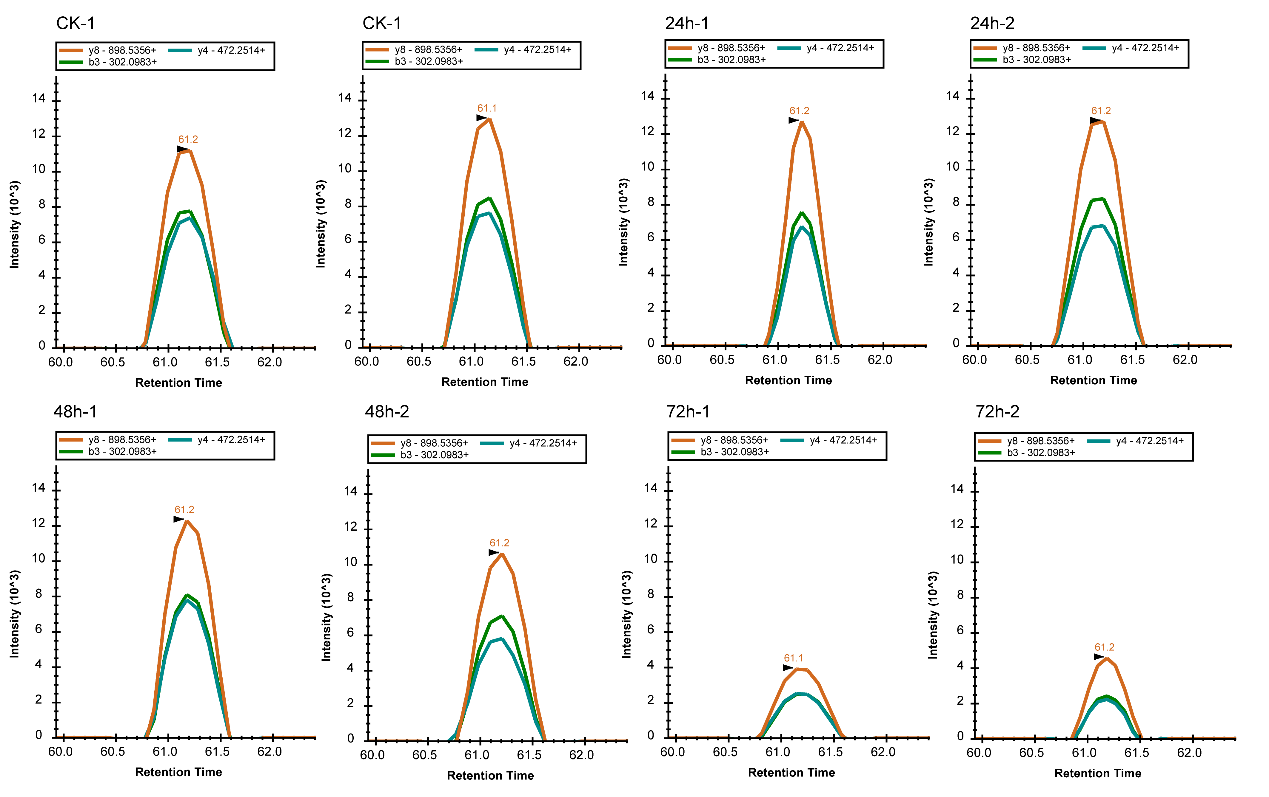


Figure S1 PRM for the abundance of the brain-specific angiogenesis inhibitor 1-associated protein 2 (B1AZ46) was determined. Graphs displaying chromatograms of fragment ions extracted from the peptide EGDLITLLVPEAR. The mass measurement error and retention time of the most intense transition are annotated above the peak.
