# Supplementary figures and images for: Comparative proteomics reveals that fatty acid metabolism is involved in myocardial adaptation to chronic hypoxic injury
Source: PLoS One. 2024 Jun 17;19(6):e0305571. doi: 10.1371/journal.pone.0305571 (PMC11182518; doi:10.1371/journal.pone.0305571)

Figure 6A

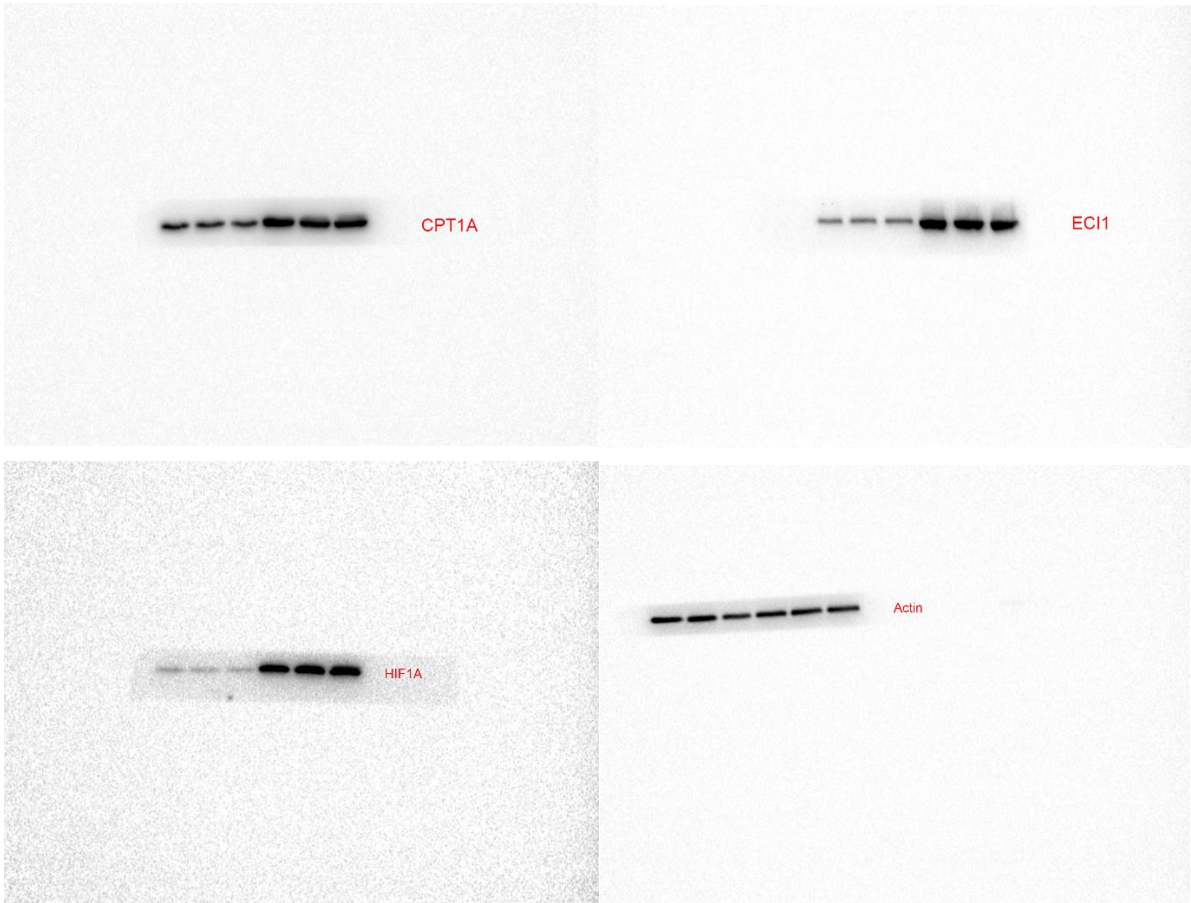

Figure 6E shCPT1A

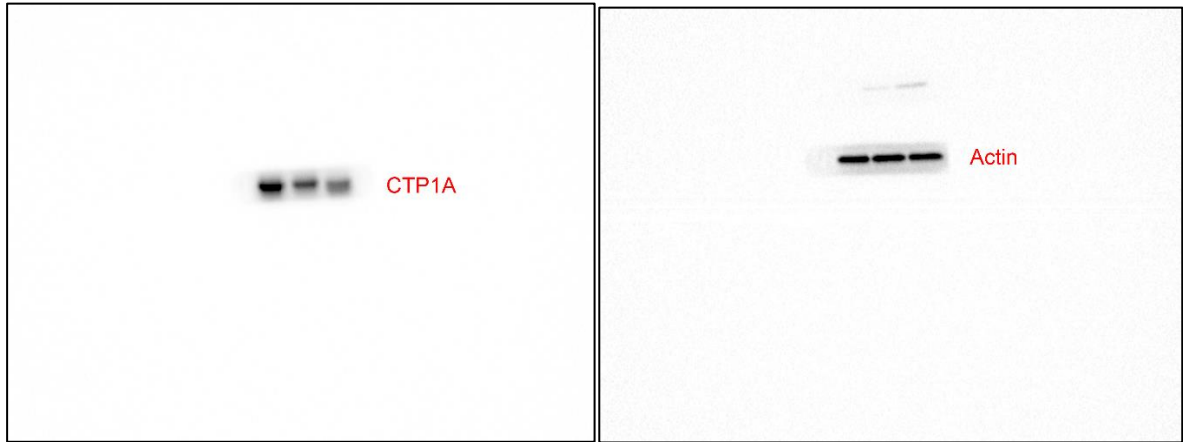

shEC11

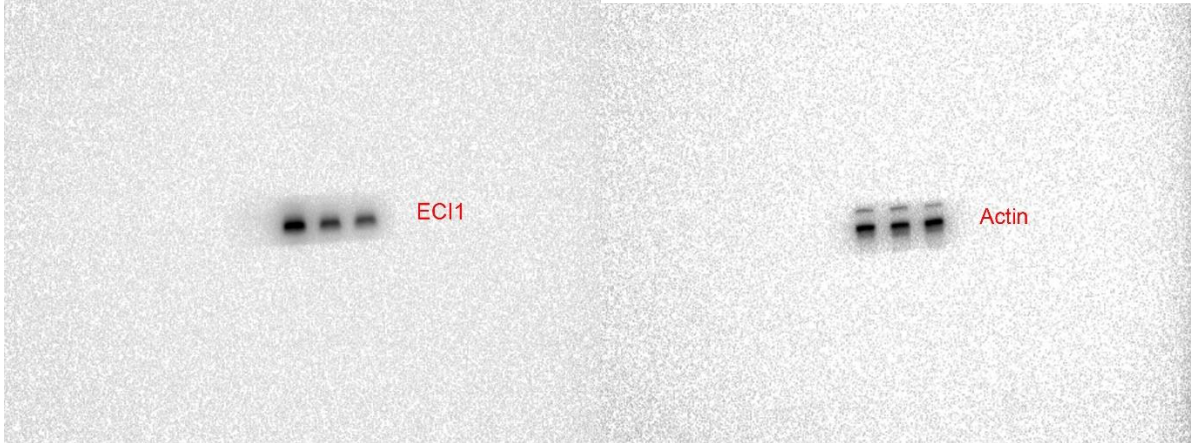

Supplement: S1 Raw images — (PDF) [file pone.0305571.s005.pdf]
