# Supplementary material for: Comparative proteomics reveals that fatty acid metabolism is involved in myocardial adaptation to chronic hypoxic injury
Source: PLoS One. 2024 Jun 17;19(6):e0305571. doi: 10.1371/journal.pone.0305571 (PMC11182518; doi:10.1371/journal.pone.0305571)
Supplement: Attachment — Submitted filename: Response to Reviewers.docx [file pone.0305571.s006.docx]

**Response to Reviewers**

**Reviewer #1**

Cyanotic congenital heart disease (CCHD) is characterized by chronic hypoxia. In this manuscript, the authors aim to utilize an in vivo hypoxia cell model to identify potential treatment targets through proteomic analysis. Their findings suggest that chronic hypoxia increases fatty acid metabolism to sustain the growth of cardiomyocytes. However, several concerns regarding data quality and methodology are outlined below:

1. Cardiomyocytes are widely known to preferentially use glucose over fatty acids as energy resources in response to chronic hypoxia (PMID: 17761770; PMID: 33663226). This contradicts the results presented in the manuscript, and the authors need to furnish additional evidence supporting their conclusion, such as conducting glucose and fatty acid uptake and oxidative experiments in response to hypoxia.

Response: We sincerely appreciate the reviewer’s constructive suggestions. In our proteome intensity dataset, 37 proteins participated into glycolysis/gluconeogenesis present varying degree increase or decline (Table S2). And the tricarboxylic acid cycle and oxidative phosphorylation is highly enriched among all quantified proteins annotating pathways (Figure 5C-5D). These results indicate that glucose is an important energy resource for cell survival in response to chronic hypoxia. Besides, we also found that proteins involved in fatty acid metabolism also enriched (Figure 5A). Moreover, several proteins increased obviously in response to chronic hypoxia (Figure 5B). Thus, we speculated that fatty acid is also important for cardiomyocyte in response to chronic hypoxia. We discussed the two published study (PMID: 17761770; PMID: 33663226) in “Discussion” section of the revised manuscript.

2. On page 17, lines 393-394, the statement that "the protein level of CD36 was not changed" does not necessarily indicate unaffected fatty acid uptake since CD36 activation involves translocation from the cytosol to the membrane. To strengthen their findings, the authors should measure membrane CD36 content and fatty acid uptake in response to hypoxia.

Response: We sincerely appreciate the reviewer’s constructive suggestions. According, we measured the expression of CD36 in cell membrane and fatty acid in response to hypoxia. As shown in Figure 6C, we found that CD36 protein mainly expressed on the cell membrane under normoxia and hypoxia. However, hypoxia did not significantly alter the expression of CD36 compared with normoxia. We found that hypoxia could remarkedly reduce the concentration of fatty acids in the cellular of MCMs with supplementing of oleic acid (Figure 6D, the revised manuscript).

3. The proteomics data with identifier PXD042055 is not accessible via Proteome Xchange Datasets, raising a concern about data transparency and accessibility.

Response: We sincerely apologize for this mistake. We reuploaded the proteomic data on Proteome Xchange Consortium with identifier PXD051081.

4. The choice of mouse cardiomyocytes (MCM) as opposed to the more widely used cardiomyocyte cell lines, H9C2 (derived from rats) and HL1 (derived from mice), necessitates validation. The authors should furnish evidence confirming the identity of the cells used as mouse cardiomyocytes.

Response: We appreciated your advice. Mouse cardiomyocytes (MCM) used in this study was confirmed by STR and authenticated as HL1 cell line. The STR genotyping report is provide in Supporting Information. So, we corrected MCM as HL1 in revised manuscript.

5. Principal Component Analysis (PCA) for the proteomic data is crucial for a comprehensive understanding of the dataset and should be provided by the authors.

Response: We appreciated your constructive suggestion. We preformed the Principal Component Analysis (PCA) and presented it in Figure 1C of the revised manuscript.

6. Blast result shows that the shRNA-targeting sequences for ECL1 list in the manuscript is shECI2-targeting sequences, but their western results show knockdown of ECI1, this inconsistency makes their results less reliability.

Response: We sincerely apologize for this careless error that we copied the sequences for ECI2 as ECI1 when we prepared the manuscript. We corrected the error in the revised manuscript.

7. The authors are urged to provide original data, particularly for Western blotting, to ensure transparency and facilitate a thorough evaluation of the results.

Response: Thank you for your advices. We provide the original data in Supporting Information.

**Reviewer #2**

Full Title: Comparative proteomics reveals that fatty acid metabolism is involved in myocardial adaptation to chronic hypoxic injury Comparative proteomics reveals that fatty acid metabolism is involved in myocardial adaptation to chronic hypoxic injury.

The authors have investigated that fatty acid metabolism is involved in myocardial adaptation to chronic hypoxic injury. The manuscript is well addressed and well written. The scientific aptitude of this work is worthy of investigation. The experiments are well designed and executed along with strong evidence of data analysis and statistics.

Comments:

1. The immunoblot experiments done from cardiomyocytes are from whole cell lysates or mitochondrial fraction?

Response: Thank you. We performed immunoblot experiments by using whole cell lysates. We indicated this issue clearly in the figure legend of the revised manuscript.

2. Is there any evidence of post transnational modification of CPT1A and HIF1-α under chronic and acute hypoxia? Please discuss briefly in the discussion section.

Response: We appreciated your suggestion. We discussed the post-translational modification of CPT1A and HIF1α in “Discussion” section of the revised manuscript.
