# Supplementary material for: Comparative proteomics reveals that fatty acid metabolism is involved in myocardial adaptation to chronic hypoxic injury
Source: PLoS One. 2024 Jun 17;19(6):e0305571. doi: 10.1371/journal.pone.0305571 (PMC11182518; doi:10.1371/journal.pone.0305571)
Supplement: Attachment — Submitted filename: Response to Reviewers.docx [file pone.0305571.s007.docx]

**Response to Reviewers**

Reviewer #1: The authors have revised the manuscript; however, there are still several comments to address, as outlined below:

1. Please provide quantification results for Figure 6C, including membrane CD36, total CD36, and the membrane/total ratio of CD36.

Response: Thank you for your advices. Accordingly, we quantified the membrane CD36, total CD36 and the membrane/total ratio of CD36 (Figure 6D-6E, the revised manuscript).

2. Regarding HL1 cells:

a) Please specify the source of the HL1 cells.

Response: Thanks for the reviewer’s comments. We sincerely apologize for the mistakes in papering this manuscript. The HL1 cells were a kind gift from Professor Jie Chao, we added the information in “Materials and Methods” of the revised manuscript.

b) Properly cultured HL1 cells typically exhibit adherent, fibroblast-like morphology. However, in Figure 1B and Figure 6C, the cell morphology appears rounded. Additionally, the low confluence and apparent suboptimal cell conditions raise concerns.

Response: As shown in followed picture, HL1 cells we cultured display adherent, fibroblast-like morphology in our tissue culture flask. In Figure 1B, the cell viability was measured by trypan blue staining after cell digestion. After staining, the cells were placed on slide, and then observed under microscope. So, the cell morphology appears rounded.

To measure the expression of CD36, the cells were seeded on glass slide, then subjected to immunofluorescent staining and confocal analysis. Due to the hydrophobicity of the glass slide and lack sufficient scaffold surface, the cells cannot attach well and spread fully. Therefore, we observed that the HL1 cells exhibit rounded shape, not fibroblast-like morphology in Figure 6C.

c) In the Materials and Methods section, the authors stated the use of DMEM to culture HL1 cells. However, optimal culture medium for HL1 cells is Claycomb medium (PMID: 9501201, PMID: 14766671), which contains numerous proteins, growth factors, and hormones crucial for maintaining cardiac myocyte phenotype and metabolism. Could the choice of culture medium have influenced the notable discoveries reported in this manuscript?

Response: We sincerely appreciate the reviewer’s comments for improving our manuscript. Indeed, we used Claycomb medium to culture HL1 cells. The HL1 cells were derived from Professor Jie Chao of the Southeast University and the corresponding media. However, our student didn’t record the correct name of this medium. During this revision, we contacted Professor Jie Chao to confirm the medium information, and corrected it in the revised manuscript.

3. On page 4, line 88, there is a duplication of "HL1"; one instance should be removed.

Response: We sincerely apologize for this careless error. Accordingly, we corrected this error.

4. On page 7, line 192, "Western Blotting" should be corrected to "Immunofluorescent."

Response: Accordingly, we corrected this error.

5. On page 7, line 201, "MCM" should be revised to "HL1."

Response: Accordingly, we corrected this error.
